# Supplementary material for: Molecular Mechanisms of the Anti-Inflammatory Effects of Epigallocatechin 3-Gallate (EGCG) in LPS-Activated BV-2 Microglia Cells
Source: Brain Sci. 2023 Apr 7;13(4):632. doi: 10.3390/brainsci13040632 (PMC10137201; doi:10.3390/brainsci13040632)
Supplement: Supplementary file 1 [file brainsci-13-00632-s001.zip › brainsci-2242855-SI.pdf]

Supplementary Table S1. PCR array analysis of cytokines/chemokines correlated to neuroinflammation and oxidative stress.

Gene of interest	Neuroimmunological purpose	References	Associated Signaling Pathway	Downregulated or Upregulated by EGCG	Fold Change t/-
Macrophage migration inhibitory factor (MIF)	Cytokine is implicated in inflammatory processes in Alzheimer's disease.	[1-3]		downregulated	Both EGCG 150 +LPS
Cytokine C-C motif ligand 2 (CCL2) aka Monocyte chemoattractant protein-1 (MCP-1)	Cytokine has a major role in amyloidogenesis in neurodegenerative disease.	[4, 5]	NF-κB	downregulated	EGCG 150 +LPS vs LPS alone 1.44-fold decrease
Interleukin 3 (IL-3)	Glycoprotein is involved in neuronal cell survival and development. It also is neuroprotective via microglia.	[6, 7]		upregulated	EGCG 150 +LPS vs LPS alone 2.33-fold increase
Interleukin 11 (IL-11)	Member of the IL-6 family of cytokines that may exhibit neurorescue capability.	[8, 9]	.	upregulated	EGCG 150 +LPS vs LPS alone 5.14-Fold increase

Interleukin 6 (IL-6)	Proinflammatory cytokine prominent in neurodegenerative disorders	[10]	NF-κB	downregulated	
Nitric Oxide (NO)	Many functions are related to inflammation, cellular survival, and neuronal immunology. It is a prominent biomarker for reactive oxygen species (ROS) and neuroinflammation.	[11, 12]	NF-κB	downregulated	
Colony-stimulating factor 2 (CSF2), aka Granulocyte-macrophage colony-stimulating factor (GM-CSF)<br><br>Colony Stimulating Factor 3 (CSF3), aka Granulocyte colony-stimulating factor (G-CSF)	Cytokine expelled by macrophages is increased in AD patients. It regulates neuroinflammation.	[13, 14]	NF-κB	upregulated	EGCG 150 +LPS vs LPS alone 8.92-fold increase (CSF3)
Tumor Necrosis Factor Subfamily, 10 (TNFS10) aka Tumor necrosis, factor-related apoptosis-inducing ligand (TRAIL)	The gene associated with the TNF superfamily exhibits proinflammatory properties in AD.	[15]	NF-κB	upregulated	EGCG 150 +LPS vs LPS alone 1.62-fold increase

Supplementary Table S2. PCR array evaluation of signaling pathways linked to neuroinflammation and oxidative stress.

Gene of interest	Neuroimmunological function	References	Signaling Pathway	Down/upregulation by EGCG	Fold Change +/-
Mitogen-activated protein kinase (MAPK) kinase kinase (MAP3k1) and Mitogen-Activated Protein Kinase 3 (MAPK3)	A key constituent of the MAPK/ERK pathway and the regulation of infalammatory processes i.e.apoptosis and lysosomal clearance mechanisms	[16]	NFkB and mTOR	downregulated	EGCG 150 +LPS and EGCG
Protein kinase c beta (PRKCB)	Enzyme correlated with mediating NO production via microglial INOS regulation	[17]	mTOR	downregulated	EGCG 150 +LPS vs LPS alone 4.03 fold decrease
Mechanistic target of rapamycin (mTOR)	Signaling pathway involved in cellular survival, nutrient sensing, autophagy, aging, and inflammation	[18]	mTOR	downregulated	EGCG 150 +LPS vs LPS alone 1.46 fold decrease
AKT Serine/Threonine Kinase 1 (AKT1)<br><br>AKT3 aka Thymoma viral proto-oncogene 3	An enzyme that is associated with insulin signaling in the brain<br><br>Serine Threonine protein kinase acting on the brain	[19-21]	mTOR/PI3k-Akt	downregulated	EGCG 150 +LPS vs. LPS alone 1.49 fold decrease (AKT3)<br><br>EGCG 150 +LPS vs. LPS alone 1.54 fold decrease (AKT1)

	may exhibit neuroprotective properties or serve to heighten ROS				
Insulin 2 (INS2)	A gene that codes for preproinsulin	[22]	mTOR	upregulated	EGCG 150 +LPS vs. LPS alone 10.42 fold increase
Phospholipase D2 (PLD2)	It is an enzyme that modulates membrane phospholipid activity, which may serve as a biomarker for aberrant protein aggregation in AD	[23]	mTOR	upregulated	EGCG 150 +LPS vs. LPS alone 1.78 fold increase
SMAD Family Member 3 (SMAD3), aka Mothers against decapentaplegic homolog 3	An effector of TGF-β signaling and enhancer of NFκB, but it also may affect aging in neurodegenerative disorders	[24]	NFκB	downregulated	EGCG 150 +LPS vs. LPS alone 1.66 fold decrease
C-C Motif Chemokine Ligand 5 (CCL5)<br><br>aka Regulated upon Activation, Normal T Cell	A chemokine that exhibits proinflammatory properties in microglia and in AD.	[25]	NFκB	downregulated	EGCG 150 +LPS vs LPS alone 1.30 fold decrease

Expressed and Presumably Secreted (RANTES)					
Nucleotide-binding oligomerization domain-containing protein 1 (NOD1)	Intracellular inspectors of inflammation serve as biomarkers for aberrant cellular action	[26]	NFκB	downregulated	EGCG 150 +LPS vs LPS alone 1.43 fold decrease
Heme Oxygenase 1 (HMOX1)	Enzymes with antioxidative action, which may act as a regulator of ferroptosis	[27]	NFκB	upregulated	EGCG 150 +LPS vs LPS alone 3.24 fold increase
Tnf receptor-associated factors (TRAF 2,3 and 5)	Constitutive elements have been implicated as biomarkers for AD, especially Traf2. They regulate autophagy, cytokine activity, and cellular development.	[28]	NFκB	downregulated	EGCG 150 +LPS vs LPS alone 1.49 fold decrease (Traf2)<br>EGCG 150 +LPS vs LPS alone 1.42 fold decrease (Traf 3)<br>EGCG 150 +LPS vs LPS alone 1.88 fold decrease (Traf 5)
Tumor Necrosis Factor (TNF)	A known inflammatory signaling pathway that involves the cytokine TNF-α, which is known to present inflammation in microglia. This gene is a biomarker for neuroinflammation in AD	[29]	NFκB	upregulated	EGCG 150 +LPS vs LPS alone 4.01 fold increase

tumor necrosis factor alpha-induced protein 3 (TNFAIP3)	This gene is shown to regulate autophagy and have anti-inflammatory properties.	[30]	NFκB	upregulated	EGCG 150 +LPS vs. LPS alone 1.41 fold increase
Toll-like receptors (TLR 1, 3, and 4) emphasis on TLR4	Intracellular ligands are a form of pattern recognition receptors (PRRs) acting to mediate immunosurveillance and aging mechanisms.	[31, 32]	NFκB and PI3k-Akt	upregulated	EGCG 150 +LPS vs. LPS alone 2.17 fold increase (TLR3)<br>EGCG 150 +LPS vs. LPS alone 1.86 fold increase and (TLR4)<br>EGCG 150 +LPS vs. LPS alone 3.05 fold increase (TLR1)
RelB Proto-Oncogene, NF-KB Subunit (RELB).	Gene is important for the proper function of microglial and neural homeostasis.	[33]	NF-κB	downregulated	EGCG 150 +LPS vs. LPS alone 1.76 fold decrease
Interleukin 1 beta<br>(IL1B)	A cytokine that is linked to IL-1 receptor activity that functions in microglial inflammation and other CNS responsibilities, i.e., functioning as a growth factor	[34]	NF-κB	downregulated	EGCG 150 +LPS vs. LPS alone 1.16 fold decrease

Nuclear Factor Kappa B2 (Nf-κB2)	The gene encodes a subunit of NFκB. NFκB is a transcription factor that is responsible for a multitude of cellular activities, but in microglia, it may regulate Tau	[35]	NFκB	downregulated	EGCG 150 +LPS vs. LPS alone 1.62 fold decrease
FBJ osteosarcoma oncogene AP-1 Transcription Factor Subunit (FOS) aka c-fos or Activator Protein 1 (AP-1)	Transcription mediators correlated with control of inflammation and microglial development. It functions to control proper brain maturation.	[36, 37]	NFκB and PI3k-akt	downregulated	EGCG 150 +LPS vs. LPS alone 2.03 fold decrease (NFκB)<br><br>EGCG 150 +LPS vs. LPS alone 1.84 fold decrease (PI3K-Akt)
Signal Transducer And Activator Of Transcription 1 (STAT1)	Important transcription factors involved in aging and neurodegenerative activity	[38]	NFκB	downregulated	EGCG 150 +LPS vs. LPS alone 2.14 fold decrease
Glutathione Peroxidases (GPX 1 and 4)	Enzymes implicated in oxidative stress regulation related to neurodegeneration.	[39]	NO	downregulated	EGCG 150 + vs. LPS alone 2.63 fold decrease (Gpx1)<br><br>EGCG 150 +LPS vs. LPS alone 2.84 fold decrease (Gpx4)

Cathepsin B (CTSB)	Lysosomal cysteine protease is active in protein aggregation in neurodegenerative ailments.	[40]	NO	downregulated	EGCG 150 +LPS vs. LPS alone 3.30 fold decrease
Immunity-related GTPase family M protein (Irgm1), aka interferon-inducible protein 1	The enzyme displays autophagic properties.	[41]	NO	downregulated	EGCG 150 +LPS vs. LPS alone 3.46 fold decrease
Nitric Oxide Synthase 1 neuronal (NOS1)	The enzyme that modulates NO activity in the brain may be a possible link to INOS	[42]	NO	downregulated	EGCG 150 +LPS vs. LPS alone 2.05 fold decrease
Phosphoprotein enriched in astrocytes 15A (elf4e<br><br>)	Protein that is known to function in apoptosis but may have autophagic regulative abilities	[43, 44]	NO	downregulated	EGCG 150 +LPS vs. LPS alone 1.57 fold decrease
Growth factor receptor bound protein 2-associated protein 1 (GAB1)	The molecule that acts to mediate cognitive function	[45]	NO	upregulated	EGCG 150 +LPS vs. LPS alone 2.15 fold increase
Cyclin-dependent kinase inhibitor 1B (CDKN1B)	Involved in cell cycle arrest and currently being studied for its presumed prevention of AD	[46]	PI3k-Akt	downregulated	EGCG 150 +LPS vs. LPS alone 2.14 fold decrease

Growth factor receptor-bound protein 2 (GRB2)	A signaling adaptor domain associated with activated Cdc42-associated kinase 1(ACK1) mediates TLR signaling. It seems to interact with amyloid beta.	[47, 48]	PI3k-Akt	downregulated	EGCG 150 +LPS vs. LPS alone 1.36 fold decrease
Insulin-like growth factor I receptor (IGF1R)	Receptors involved in managing IGF-1, which controls aging and neuroinflammation.	[49, 50]	PI3k-Akt	downregulated	EGCG 150 +LPS vs. LPS alone 1.55 fold decrease
V-raf-leukemia viral oncogene 1 (RAF1)	An initiator of Extracellular signal-regulated kinase (ERK) signaling that is expressed highly in the AD brain	[51]	PI3k-Akt	downregulated	EGCG 150 +LPS vs LPS alone 1.94 fold decrease
Eukaryotic translation initiation factor 4E (ELF4E)	Translational modulator altered in AD.	[52]	PI3K-AKT	upregulated	LPS alone vs. control 8.64 fold increase<br><br>Both EGCG 150 +LPS vs. LPS alone 3.45 fold increase

Son of sevenless homolog 1 (Drosophila) (SOS1)	A guanine nucleotide exchange factor (GEF) that interacts with RAS	[53]	PI3k-Akt	downregulated	EGCG 150 +LPS vs. LPS alone 1.70 fold decrease
Glucuronidase, beta (GUSB)	The gene that codes for the processing of β glucuronidase, which is involved in the lysosomal breakdown of large cells glycosaminoglycans (GAGs), May be connected to AD.	[54]	PI3k-Akt	downregulated	EGCG 150 +LPS vs. LPS alone 1.42 fold decrease
The regulatory-associated protein of MTOR, complex 1 (RPTOR)	The regulative gene that modulates the nutrient-sensing capabilities of mTORC1	[55]	mTOR	downregulated	EGCG 150 +LPS vs. LPS alone 1.72 fold decrease
Protein kinase, AMP-activated, beta 2 non-catalytic subunit (PRKAB2)	A gene associated with AMPK in regulating energy and cellular bioenergetics along with mTORC1	[56]	mTOR	downregulated	EGCG 150 +LPS vs. LPS alone 1.516 fold decrease
Cytochrome b-245, alpha polypeptide (CYBA)	The gene that encodes for a unit associated with the nicotinamide adenine dinucleotide phosphate oxidase (NADPH) complex that produces superoxide	[57-59]	NO	downregulated	EGCG 150 +LPS vs. LPS alone 3.18 fold decrease

	dismutase (SOD) and is involved in phagocytosis				
Hepsin (HPN)	A transmembrane serine protease involved in many cellular process but may have a role in activating matrix metalloproteinases.	[60-62]	NO	downregulated	EGCG 150 +LPS vs LPS alone 3.08 fold decrease
Growth Arrest and DNA Damage Inducible Protein (GADD45A)	A thoroughly studied protein involved in aging, molecular stress control, DNA repair, and cellular development	[63-66]	NO	downregulated	EGCG 150+ LPS vs LPS alone 2.42 fold decrease

References

1. Calandra, T. and T. Roger, *Macrophage migration inhibitory factor: a regulator of innate immunity.* Nat Rev Immunol, 2003. **3**(10): p. 791–800.
2. Nasiri, E., et al., *Key role of MIF-related neuroinflammation in neurodegeneration and cognitive impairment in Alzheimer's disease.* Mol Med, 2020. **26**(1): p. 34.
3. Xuan, W., et al., *Dualistic roles and mechanistic insights of macrophage migration inhibitory factor in brain injury and neurodegenerative diseases.* J Cereb Blood Flow Metab, 2022: p. 271678x221138412.
4. Bose, S. and J. Cho, *Role of chemokine CCL2 and its receptor CCR2 in neurodegenerative diseases.* Arch Pharm Res, 2013. **36**(9): p. 1039-50.
5. Joly-Amado, A. et al., *CCL2 Overexpression in the Brain Promotes Glial Activation and Accelerates Tau Pathology in a Mouse Model of Tauopathy.* Front Immunol, 2020. **11**: p. 997.
6. Zambrano, A. et al., *Interleukin-3 prevents neuronal death induced by amyloid peptide.* BMC Neurosci, 2007. **8**: p. 82.
7. Klein, R.S., *Astrocyte interleukin-3 preps microglia.* Trends Immunol, 2021. **42**(11): p. 937-939.
8. Fung, K.Y., et al., *Emerging roles for IL-11 in inflammatory diseases.* Cytokine, 2022. **149**: p. 155750.
9. Zhang, B. et al., *Interleukin-11 treatment protected against cerebral ischemia/reperfusion injury.* Biomed Pharmacother, 2019. **115**: p. 108816.
10. Lyra, ESNM, et al., *Proinflammatory interleukin-6 signaling links cognitive impairments and peripheral metabolic alterations in Alzheimer's disease.* Transl Psychiatry, 2021. **11**(1): p. 251.
11. Chachlaki, K. and V. Prevot, *Nitric oxide signalling in the brain and its control of bodily functions.* Br J Pharmacol, 2020. **177**(24): p. 5437-5458.
12. Streyczek, J., et al., *Turmeric Extract (Curcuma longa) Mediates Antioxidative Effects by Reduction of Nitric Oxide, iNOS Protein-, and mRNA-Synthesis in BV2 Microglial Cells.* Molecules, 2022. **27**(3).
13. Chitu, V., et al., *Microglial Homeostasis Requires Balanced CSF-1/CSF-2 Receptor Signaling.* Cell Rep, 2020. **30**(9): p. 3004-3019.e5.
14. Spath, S., et al., *Dysregulation of the Cytokine GM-CSF Induces Spontaneous Phagocyte Invasion and Immunopathology in the Central Nervous System.* Immunity, 2017. **46**(2): p. 245-260.
15. Frenkel, D., *A new TRAIL in Alzheimer's disease therapy.* Brain, 2015. **138**(Pt 1): p. 8-10.

16. Moens, U., S. Kostenko, and B. Sveinbjørnsson, *The Role of Mitogen-Activated Protein Kinase-Activated Protein Kinases (MAPKAPKs) in Inflammation.* Genes (Basel), 2013. **4**(2): p. 101-33.
17. Wen, J., R. Ribeiro, and Y. Zhang, *Specific PKC isoforms regulate LPS-stimulated iNOS induction in murine microglial cells.* J Neuroinflammation, 2011. **8**: p. 38.
18. Sabatini, D.M., *Twenty-five years of mTOR: Uncovering the link from nutrients to growth.* Proc Natl Acad Sci U S A, 2017. **114**(45): p. 11818-11825.
19. Gabbouj, S., et al., *Altered Insulin Signaling in Alzheimer's Disease Brain - Special Emphasis on PI3K-Akt Pathway.* Front Neurosci, 2019. **13**: p. 629.
20. Polytarchou, C., et al., *Akt3 induces oxidative stress and DNA damage by activating the NADPH oxidase via phosphorylation of p47(phox).* Proc Natl Acad Sci U S A, 2020. **117**(46): p. 28806-28815.
21. DuBois, J.C., et al., *Akt3-Mediated Protection Against Inflammatory Demyelinating Disease.* Front Immunol, 2019. **10**: p. 1738.
22. Ardestani, A., et al., *mTORC1 Signaling: A Double-Edged Sword in Diabetic β Cells.* Cell Metab, 2018. **27**(2): p. 314-331.
23. Kosicek, M. and S. Hecimovic, *Phospholipids and Alzheimer's disease: alterations, mechanisms and potential biomarkers.* Int J Mol Sci, 2013. **14**(1): p. 1310-22.
24. Tichauer, J.E., et al., *Age-dependent changes on TGFβ1 Smad3 pathway modify the pattern of microglial cell activation.* Brain Behav Immun, 2014. **37**: p. 187-96.
25. Zuena, A.R., et al., *Chemokines in Alzheimer's Disease: New Insights Into Prokineticins, Chemokine-Like Proteins.* Front Pharmacol, 2019. **10**: p. 622.
26. Mukherjee, T., et al., *NOD1 and NOD2 in inflammation, immunity and disease.* Arch Biochem Biophys, 2019. **670**: p. 69-81.
27. Campbell, N.K., H.K. Fitzgerald, and A. Dunne, *Regulation of inflammation by the antioxidant haem oxygenase 1.* Nat Rev Immunol, 2021. **21**(7): p. 411-425.
28. Culpan, D., et al., *TNFR-associated factor-2 (TRAF-2) in Alzheimer's disease.* Neurobiol Aging, 2009. **30**(7): p. 1052-60.
29. Oh, S.L., et al., *Alzheimer's Disease Blood Biomarkers Associated With Neuroinflammation as Therapeutic Targets for Early Personalized Intervention.* Front Digit Health, 2022. **4**: p. 875895.
30. Chen, J., et al., *TNFAIP3 ameliorates the degeneration of inflammatory human nucleus pulposus cells by inhibiting mTOR signaling and promoting autophagy.* Aging (Albany NY), 2020. **12**(23): p. 24242-24254.
31. Shaw, A.C., et al., *dysregulation of human Toll-like receptor function in aging.* Ageing Res Rev, 2011. **10**(3): p. 346-53.
32. Fiebich, B.L., et al., *Role of Microglia TLRs in Neurodegeneration.* Front Cell Neurosci, 2018. **12**: p. 329.
33. Mockenhaupt, K., A. Gonsiewski, and T. Kordula, *RelB and Neuroinflammation.* Cells, 2021. **10**(7).
34. Liu, X. and N. Quan, *Microglia and CNS Interleukin-1: Beyond Immunological Concepts.* Front Neurol, 2018. **9**: p. 8.
35. Wang, C., et al., *Microglial NF-κB drives Tau spreading and toxicity in a mouse model of tauopathy.* Nat Commun, 2022. **13**(1): p. 1969.
36. Holtman, I.R., D. Skola, and CK Glass, *Transcriptional control of microglia phenotypes in health and disease.* J Clin Invest, 2017. **127**(9): p. 3220-3229.
37. Velazquez, F.N., B.L. Caputto, and F.D. Boussin, *c-Fos importance for brain development.* Aging (Albany NY), 2015. **7**(12): p. 1028-9.
38. Butturini, E., et al., *STAT1 drives M1 microglia activation and neuroinflammation under hypoxia.* Arch Biochem Biophys, 2019. **669**: p. 22-30.
39. Ansari, M.A. and S.W. Scheff, *Oxidative stress in the progression of Alzheimer disease in the frontal cortex.* J Neuropathol Exp Neurol, 2010. **69**(2): p. 155-67.
40. Tsujimura, A., et al., *Lysosomal enzyme cathepsin B enhances the aggregate forming activity of exogenous α-synuclein fibrils.* Neurobiol Dis, 2015. **73**: p. 244-53.
41. He, S., et al., *Immune-related GTPase M (IRGM1) regulates neuronal autophagy in a mouse model of stroke.* Autophagy, 2012. **8**(11): p. 1621-7.
42. Mattila, J.T. and A.C. Thomas, *Nitric oxide synthase: non-canonical expression patterns.* Front Immunol, 2014. **5**: p. 478.
43. Fiory, F., et al., *Frontiers: PED/PEA-15, a multifunctional protein controlling cell survival and glucose metabolism.* Am J Physiol Endocrinol Metab, 2009. **297**(3): p. E592-601.
44. Böck, B.C., et al., *The PEA-15 protein regulates autophagy via activation of JNK.* J Biol Chem, 2010. **285**(28): p. 21644-54.
45. Lu, N.N., et al., *Cholinergic Grb2-Associated-Binding Protein 1 Regulates Cognitive Function.* Cereb Cortex, 2018. **28**(7): p. 2391-2404.
46. Malhotra, N., R. Gupta, and P. Kumar, *Pharmacological relevance of CDK inhibitors in Alzheimer's disease.* Neurochem Int, 2021. **148**: p. 105115.
47. Jing, L., et al., *ACK1 Contributes to the Pathogenesis of Inflammation and Autoimmunity by Promoting the Activation of TLR Signaling Pathways.* Front Immunol, 2022. **13**: p. 864995.
48. Raychaudhuri, M. and D. Mukhopadhyay, *Grb2-mediated alteration in the trafficking of AbetaPP: insights from Grb2-AICD interaction.* J Alzheimers Dis, 2010. **20**(1): p. 275-92.

49. Gubbi, S., et al., *40 YEARS of IGF1: IGF1: the Jekyll and Hyde of the aging brain.* J Mol Endocrinol, 2018. **61**(1): p. T171-t185.
50. Labandeira-Garcia, J.L., et al., *Insulin-Like Growth Factor-1 and Neuroinflammation.* Front Aging Neurosci, 2017. **9**: p. 365.
51. Mei, M., et al., *Distribution, levels and phosphorylation of Raf-1 in Alzheimer's disease.* J Neurochem, 2006. **99**(5): p. 1377-88.
52. Ghosh, A., et al., *Alzheimer's disease-related dysregulation of mRNA translation causes key pathological features with ageing.* Transl Psychiatry, 2020. **10**(1): p. 192.
53. Johnson, D.S. and Y.H. Chen, *Ras family of small GTPases in immunity and inflammation.* Curr Opin Pharmacol, 2012. **12**(4): p. 458-63.
54. Hwang, J., et al., *The Role of Lysosomes in a Broad Disease-Modifying Approach Evaluated across Transgenic Mouse Models of Alzheimer's Disease and Parkinson's Disease and Models of Mild Cognitive Impairment.* Int J Mol Sci, 2019. **20**(18).
55. Kim, D.H., et al., *mTOR interacts with raptors to form a nutrient-sensitive complex that signals to the cell growth machinery.* Cell, 2002. **110**(2): p. 163-75.
56. Sukumaran, A., K. Choi, and B. Dasgupta, *Insight on Transcriptional Regulation of the Energy Sensing AMPK and Biosynthetic mTOR Pathway Genes.* Front Cell Dev Biol, 2020. **8**: p. 671.
57. Shimohama, S., et al., *Activation of NADPH oxidase in Alzheimer's disease brains.* Biochem Biophys Res Commun, 2000. **273**(1): p. 5-9.
58. Ma, MW, et al., *NADPH oxidase in brain injury and neurodegenerative disorders.* Mol Neurodegener, 2017. **12**(1): p. 7.
59. Shahraz, A., et al., *Phagocytosis-related NADPH oxidase 2 subunit gp91phox contributes to neurodegeneration after repeated systemic challenge with lipopolysaccharides.* Glia, 2021. **69**(1): p. 137-150.
60. Li, S., et al., *Hepsin: a multifunctional transmembrane serine protease in pathobiology.* Febs j, 2021. **288**(18): p. 5252-5264.
61. Wilkinson, D.J., et al., *The serine proteinase hepsin is an activator of pro-matrix metalloproteinases: molecular mechanisms and implications for extracellular matrix turnover.* Sci Rep, 2017. **7**(1): p. 16693.
62. Zipfel, P., et al., *Matrix Metalloproteinases as New Targets in Alzheimer's Disease: Opportunities and Challenges.* J Med Chem, 2020. **63**(19): p. 10705-10725.
63. Sultan, F.A. and J.D. Sweatt, *The role of the Gadd45 family in the nervous system: a focus on neurodevelopment, neuronal injury, and cognitive neuroepigenetics.* Adv Exp Med Biol, 2013. **793**: p. 81-119.
64. Moskalev, A.A., et al., *Gadd45 proteins: relevance to aging, longevity, and age-related pathologies.* Ageing Res Rev, 2012. **11**(1): p. 51-66.
65. Zaidi, M.R. and D.A. Liebermann, *Gadd45 in Senescence.* Adv Exp Med Biol, 2022. **1360**: p. 109-116.
66. Torp, R., et al., *GADD45 is induced in Alzheimer's disease and protects against apoptosis in vitro.* Neurobiol Dis, 1998. **5**(4): p. 245-52.
